# Effect of the degree of ischaemic injury and reoxygenation time on the type of myocardial cell death in man: role of caspases

**DOI:** 10.1186/1472-6793-5-14

**Published:** 2005-08-19

**Authors:** Hunaid A Vohra, Manuel Galiñanes

**Affiliations:** 1 Cardiac Surgery Unit, Department of Cardiovascular Sciences, University of Leicester Leicester, UK

## Abstract

**Background:**

The importance of apoptosis in the injury sustained by the human myocardium during ischaemia and reoxygenation and the underlying mechanisms remain unclear. To quantify apoptosis and necrosis induced by simulated ischaemia/reoxygenation in the human atrial myocardium, free-hand sections of right atrial appendage (n = 8/group) were subjected to 90 minutes simulated ischaemia followed by 2, 8 and 24 hours reoxygenation.

**Results:**

Apoptosis, as assessed by TUNEL, was greater than necrosis after 90 minutes simulated ischaemia and 2 hours reoxygenation (35.32 ± 3.22% vs 13.55 ± 1.3%; p < 0.05) but necrosis was greater than apoptosis by 24 hours reoxygenation (45.20 ± 2.75% vs 4.82 ± 0.79%; p < 0.05). Total caspase activation was similar after 90 minutes simulated ischaemia followed by 2 hours and 24 hours reoxygenation (515270 ± 99570 U vs 542940 ± 95216 U; p = NS). However, caspase-3 like activation was higher at 2 hours than at 24 hours reoxygenation (135900 ± 42200 U vs 54970 ± 19100 U; p < 0.05). Inhibition of caspase-3 by z.DEVD.fmk (70 nM) almost completely abolished apoptosis from 23.26 ± 2.854% to 0.73 ± 0.28 % (p < 0.05), without affecting necrosis.

**Conclusion:**

Cell death by apoptosis and necrosis in the human myocardium subjected to simulated ischaemia/reoxygenation depends on the degree of the ischaemic insult and have a different time-course with apoptosis happening early during reoxygenation and necrosis becoming more important later. Importantly, the apoptosis induced by simulated ischaemia/reoxygenation is mainly mediated by activation of caspase-3 but it does not affect necrosis.

## Background

Ischaemia/reoxygenation of the heart induces apoptosis and necrosis [1-4]; however, the actual contribution of these two forms of cell death to ischaemia/reoxygenation injury and their time-course have not been established in the human myocardium and remains controversial in experimental animal models. Kajstura et al [3] have reported that apoptosis begins in rat ischaemic myocardium either after a prolonged period of permanent ischaemia or during a much shorter period of ischaemia followed by reperfusion whereas others [1,2] have shown that although apoptosis may be initiated during ischaemia, its detection is increased and may well be accelerated during reperfusion. On the other hand, studies in a dog model of coronary artery ligation have shown that necrosis, quantified histologically, develops rapidly after ischaemia and is directly proportional to the ischaemic time [5], although, there is evidence in the literature suggesting that necrosis may also result secondary to reperfusion injury [4]. Furthermore, it has also been suggested that apoptosis may switch to necrosis below certain critical levels of ATP [6,7].

The mechanisms of ischaemia/reoxygenation injury have been extensively investigated, however its pathophysiology is complex and the involvement and importance of various pathways such as the caspases remains unclear.

The aims of the present studies were: (i) to investigate the degree and the time-course of apoptosis and necrosis sustained during ischaemia and reoxygenation of the human myocardium and (ii) to examine the role of caspase activation. Here we have demonstrated that the type of cell death induced by simulated ischaemia/reoxygenation of the human myocardium depends on the ischaemic insult with apoptosis happening early during reoxygenation and necrosis becoming more important later. In addition, the present studies have shown the apoptosis induced by simulated ischaemia/reoxygenation is mainly mediated by activation of caspace-3 but that it does not affect necrosis.

## Results

### Study 1: Effect of the intensity of ischaemic injury and the time of reoxygenation

#### (i) Apoptosis and necrosis

**Figure 1:**
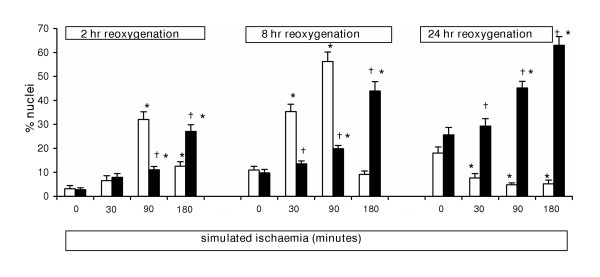
Percentage of apoptosis (empty columns) and necrosis (solid columns) in right atrial muscles subjected to various periods of simulated ischaemia followed by various periods of reoxygenation. The columns represent the mean of 8 experiments and the bars represent the SEM. *p < 0.05 vs aerobic control. †p < 0.05 vs apoptosis.

**Figure 2:**
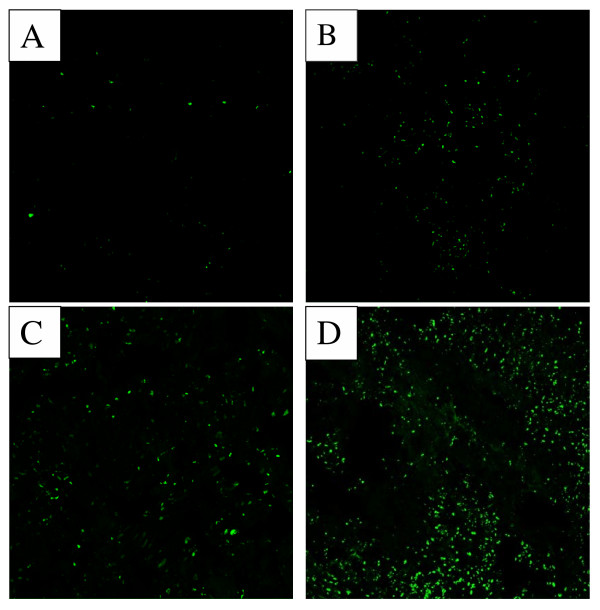
Representative images for apoptosis (TUNEL) in the following protocols: aerobic control (A); 30, 90 and 180 minutes of simulated ischaemia followed by 120 minutes of reoxygenation (B, C and D, respectively).

**Figure 3:**
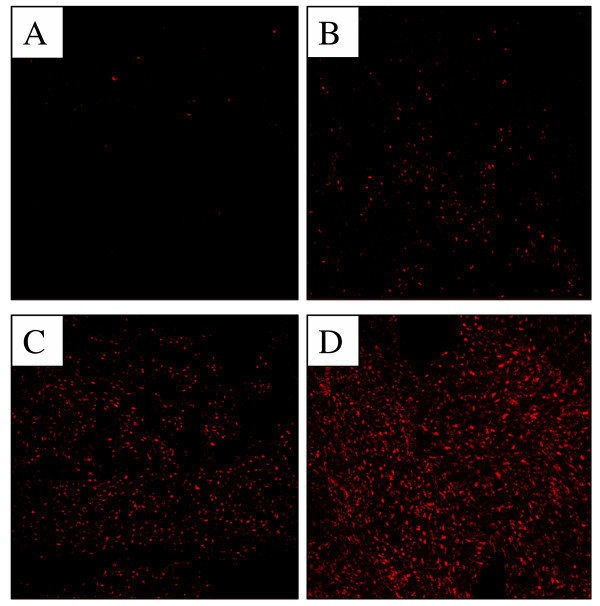
Representative images for necrosis (propidium iodide) in the following protocols: aerobic control (A); 30, 90 and 180 minutes of simulated ischaemia followed by 120 minutes of reoxygenation (B, C and D respectively).

Figure 1 shows a low degree of apoptosis and necrosis in muscles aerobically incubated for 2 hours (3.2 ± 1.3% and 2.8 ± 0.8%, respectively) and further increases of the two forms of cell death with the extension of aerobic incubation to 8 and 24 hours. As expected, the degree of necrosis increased with extension of ischaemia, however apoptosis was greater than necrosis (32.0 ± 3.2% vs 10.7 ± 1.9; p < 0.05) after 90 minutes of ischaemia and two hours of reoxygenation and the reverse was seen after 180 minutes of ischaemia (12.6 ± 1.9% vs 27.1% ± 2.8%; p < 0.05). A similar cell death pattern was observed after 8 hours of reoxygenation although the extent of apoptosis and necrosis was greater than after 2 hours of reoxygenation. After 24 hours of reoxygenation, necrosis also increased with the extension of ischaemia and it was greater than after 2 and 8 hours of reoxygenation, but necrosis was significantly more important than apoptosis. Figures 2 and 3 depict representative images of fluorescent apoptotic and necrotic nuclei.

#### (ii) MTT reduction

**Figure 4:**
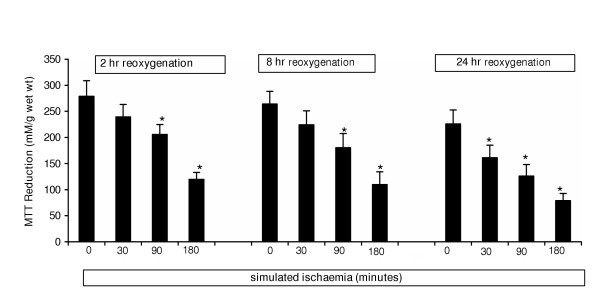
MTT reduction of right atrial muscles subjected to various periods of simulated ischaemia followed by various periods of reoxygenation. The columns represent the mean of 8 experiments and the bars represent the SEM. *p < 0.05 vs aerobic control.

Figure 4 shows that there was a decrease in MTT reduction with increasing periods of ischaemia and that, in contrast with the assessment of apoptosis and necrosis by the TUNEL assay, it was not significantly influenced by increasing the period of reoxygenation.

#### (iii) CK release

Table 1 demonstrates that the CK release exhibited the lowest values in the aerobic control groups and that there was an increase in CK release with increasing ischaemia. The mean CK release values during the first two hours of reoxygenation were similar in the groups with identical ischaemic time suggesting that ischaemic injury was of the same degree in the three reoxygenation protocols

#### (iv) Caspase activity

**Figure 5:**
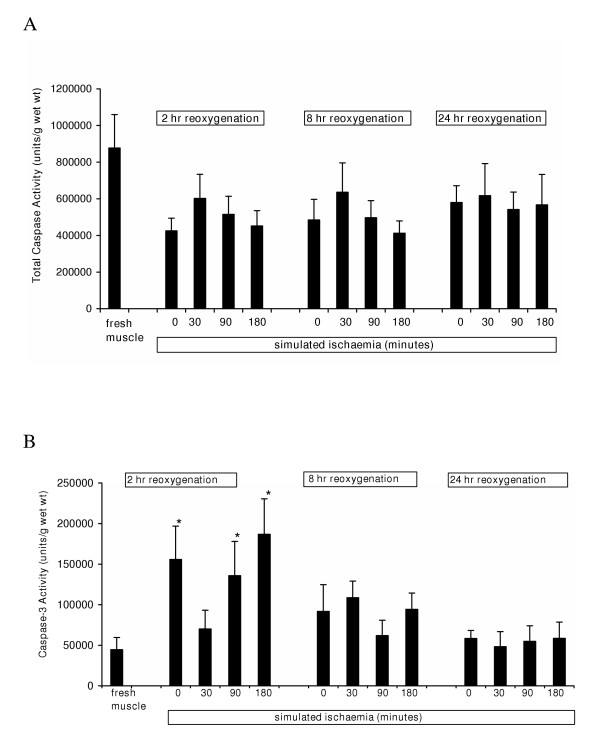
Total caspase activity (A) and caspase-3-like activity (B) in right atrial muscles subjected to various periods of simulated ischaemia followed by various periods of reoxygenation. The columns represent the mean of 8 experiments and the bars represent the SEM. *p < 0.05 vs fresh muscle

Figure 5A shows that there was no significant difference in the total caspase activation irrespective of the time of ischaemia or reoxygenation amongst the groups and that, unexpectedly, the highest values corresponded to the fresh tissue. This activity decreased after 30 minutes of aerobic equilibration. The physiological meaning of this transient increase in total caspase activity is unclear but was not associated with greater caspase-3 activation (see below) and was not translated into a rise in apoptosis.

Figure 5B demonstrates that caspase-3-like activation was significantly increased after 2 hours of aerobic incubation when compared to the mean values in the fresh muscles and that although activity increased with the extension of the ischaemic time, values were not greater than the ones seen in the aerobic control group. Importantly, by 8 and 24 hours of reoxygenation the levels of caspase-3-like activation had decreased to levels close to the values observed in the fresh muscles irrespective of the periods of ischaemia.

### Study 2: The role of caspase activation in cell death

#### (i) Inhibition of caspase-3 activity

**Figure 6:**
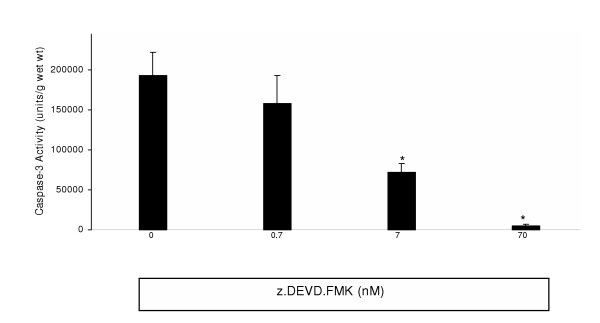
Dose-response effect of the caspase-3 inhibitor z.DEVD.FMK on caspase-3 activitiy of atrial muscles subjected to 90 minutes of simulated ischaemia followed by 120 minutes of reoxygenation. Data are expressed as the mean of 4 experiments and the bars represent the SEM. *p < 0.05 vs group without inhibitor.

Figure 6 shows that following 90 minutes of simulated ischaemia and 2 hours of reperfusion there was a dose-dependent reduction in caspase-3 activity with increasing concentration of z.DEVD.FMK, and that activity was almost completely abolished with 70 nM concentration of the inhibitor.

#### (ii) Apoptosis and Necrosis

**Figure 7:**
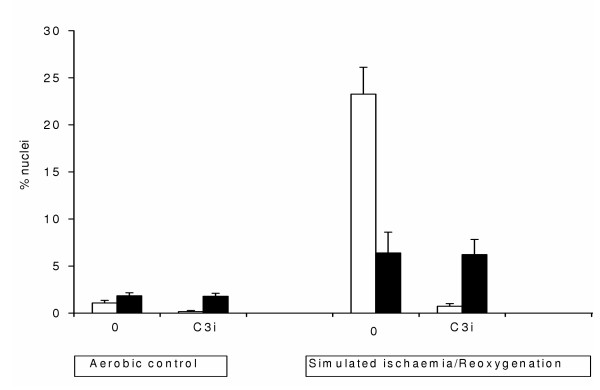
Effect of caspase-3 inhibition (C3i) with z.DEVD.FMK (70 nM) on apoptosis (empty columns) and necrosis (solid columns) in right atrial muscles subjected to 90 minutes of simulated ischaemia and 2 hours of reoxygenation. The columns represent the mean of 6 experiments and the bars represent the SEM. *p < 0.05 vs corresponding group without caspase-3 inhibitor.

Figure 7 shows the percentage of apoptosis and necrosis in atrial tissue after SI/R in the absence and presence of the caspase-3 inhibitor z.DEVD.FMK (70 nM). Caspase-3 inhibition significantly reduced apoptosis in the muscles aerobically incubated and resulted in almost complete abolition of apoptosis, from 23.3 ± 2.8% to 0.7 ± 0.3% (p < 0.05), in the muscles subjected to simulated ischaemia/reoxygenation. Interestingly, z.DEVD.FMK did not influence the degree of necrosis.

#### (iii) MTT Reduction

Table 2 shows that there was no significant change in MTT reduction with the addition of z.DEVD.FMK in the muscles aerobically incubated and those subjected to simulated ischaemia/reoxygenation. Since, as seen above, apoptosis was almost abolished whereas necrosis was unaffected by caspase-3 inhibition, these results suggest that the observed changes in MTT reduction are not a reflection of apoptosis.

#### (iv) CK release

Table 2 also shows that the addition of z.DEVD.FMK did not affect the CK release of the aerobically incubated muscle and that the increase in CK release induced by 90 minutes of simulated ischaemia and 2 hours reoxygenation was unchanged, this suggesting that, as seen with MTT reduction, changes in CK release are not a reflection of apoptosis.

## Discussion

The present studies have demonstrated that, depending on the ischaemic insult, apoptosis may be the predominant form of cell death in the human myocardium during the first 8 hours of reoxygenation, so that apoptosis is more important than necrosis when the ischaemic period is ≤ 90 minutes but the reverse is true after 180 minutes of ischaemia; however, by 24 hours of reperfusion cell death by apoptosis has subsided and necrosis, that also depends on the degree of ischaemia, becomes the leading cause of cell death. The implications of these findings for the understanding of the pathophysiology of ischaemia/reoxygenation injury are discussed below.

### Effect of the degree of ischaemic injury and the duration of reoxygenation on the type of cell death

Our study is the first to report on the time-course of cell death by apoptosis and necrosis induced by ischaemia and reoxygenation of the human myocardium. The demonstration that apoptosis follows a bell-shaped profile, increasing with the duration of ischaemia up to 90 minutes and then decreasing by 180 minutes of ischaemia, is novel. Previous experimental studies could not show this response because they used limited time-periods of ischaemia [1,8,9]. The finding that apoptosis increases with the duration of reoxygenation from 2 to 8 hours is supported by observations in the dog heart subjected to 60 minutes of ischaemia and reperfused for 6, 24, 48 and 72 hours [9]. However, whilst our study showed a decline in apoptosis when the myocardium was reoxygenated for 24 hours, the latter study [9] reported a progressive increase in apoptosis over the 72 hours of reperfusion. The reason for the differing results of the two studies is unclear but the use of different species (e.g., man versus dog) and experimental preparations (eg, in vitro versus in vivo) may be, at least in part, responsible.

The present studies have also shown that, as expected, the degree of cell death by necrosis is directly proportional to the severity of ischaemia. But, in addition, they have demonstrated that necrosis gradually increases with the duration of reoxygenation, which is consistent with the observation that necrosis is a dynamic process that continues over a period of at least 24 hours of reperfusion [9]. It should be mentioned, however, that the degree of tissue injury as assessed by the reduction of MTT was not increased with the duration of reoxygenation. This apparent contradiction between the results obtained with the propidium iodide staining and MTT reduction is not clear, but it is possible that while the former reflects the extent of necrosis alone, the latter may represent other forms of cell death. The lack of agreement of these two assays highlights the importance of using more than one index to assess tissue injury.

### Role of caspase activation on ischaemia/reoxygenation-induced cell death

The similar mean values of total caspase activity seen after all studied ischaemic and reoxygenation periods may suggest that this pathway may not be an important mechanism of cell death in the human myocardium or, more likely, that only specific caspases may take part in this process, the changes of which may not be of sufficient magnitude to significantly alter the whole pool of caspases. Our finding that caspase-3 activity is increased by the degree of ischaemic insult would support the latter hypo-thesis. The rapid dissipation of caspase-3 activity with the extension of the reoxygenation period suggests that this enzyme may be activated for a limited time period and that, therefore, the time points investigated in our studies do not provide a complete time-course of changes in enzyme activity. A participation of caspases in apoptosis has also been observed in experimental animal studies [10], with elevation of caspase-3 activity during the early reperfusion period [11,12]. It is of interest to note that in the present studies total caspase activity, but not caspase-3 activity, was elevated in fresh tissue, a finding that may be explained by the handling and mechanical injury sustained during the sectioning of the muscle.

The almost complete abolition of apoptosis by the specific caspase-3 inhibitor z.DEVD.FMK seen in our studies demonstrates that the induction of apoptosis by ischaemia/reoxygenation in the human myocardium is caspase-3 dependent. This finding in man is supported by in vitro and in vivo animal experimental studies [13,14] in which caspase-3 inhibition attenuated apoptosis and reduced reperfusion injury, all suggesting that caspase-3 activation is an obligatory step in the signal transduction pathway of apoptosis induced by ischaemia/reoxygenation. However, necrosis was unaffected by caspase-3 inhibition, which agrees with results reported in adult rat ventricular myocytes [12], although it has been observed that in cancer cells caspase inhibitors also retard necrosis in an in vitro model of chemical hypoxia [7].

Although these studies did not address the mechanism of caspase-3 activation, it has been previously shown that caspases can be activated by oxidative stress [15], an important element of ischaemia/reoxygenation injury. Furthermore, the apoptosis elicited by oxidative stress can be reduced by the opening of mitoK_ATP _channels [16] and the apoptosis and caspase-3 activation induced by ischaemia/reoxygenation can be reduced by overexpression of the heat shock proteins HSP-60 and HSP-10 [17]. It is clear that more investigation is required to elucidate the signalling pathway leading to activation of caspase-3 during ischaemia/reoxygenation.

### Study limitations

In the present studies the human atrial tissue was used and therefore any extrapolation to the ventricular myocardium should be made with caution. Thus, for example, K_ATP _channels that play a role in ischaemic injury exist in both atrium and ventricle [18], although their distribution differs in the two cardiac chambers [19]. Another potential limitation of these studies is that ischaemia and reperfusion were simulated by artificial means instead of using arterial occlusion and release, however, the avoidance of the confounding effects of collateral flow with our preparation could be advantageous. The model of simulated ischaemia and reoxygenation used in the present studies was characterised in our laboratory [20] and subsequently has been used for the investigation of the mechanism of ischaemic and pharmacological preconditioning in man [21-25].

## Conclusion

Here we have shown that cell death by apoptosis and necrosis in the human myocardium subjected to ischaemia and reoxygenation depends on the degree of the ischaemic insult and have a different time-course with apoptosis happening early during reoxygenation and necrosis becoming more important later. They also have shown that caspase-3 activation plays a critical role in apoptosis but that it does not affect necrosis.

## Methods

### Patients

The right atrial appendage from patients undergoing elective coronary artery bypass graft surgery was retrieved at the time of the right atrial cannulation. For this, local ethical approval and patients' informed consent was obtained. Patients with atrial fibrillation, poor ejection fraction (EF < 30%), and those with diabetes and taking potassium channel openers (nicorandil or diazoxide) were excluded.

### Experimental preparation and solutions

The sectioning of the atrial muscle and the preparation of simulated ischaemia/reoxygenation have been previously described [20]. Briefly, the appendage was mounted onto an ice cooled ground glass plate with the epicardial surface face down and then sliced freehand with surgical skin graft blades (Shwann-Morton, UK) to a thickness of between 300 and 500 μm. Muscle sections weighing between 30–50 mg were then transferred to conical flasks (25 ml Erlenmeyer flasks, Duran, Astell Scientific, Sidcup, Kent, UK) containing 10 ml of oxygenated buffered solution. Following this, the flasks were placed in a shaking water bath maintained at 37°C. The oxygenation of the incubation medium was maintained by a continuous flow of 95% O_2_/5%CO_2 _gas mixture to obtain a pO_2 _between 25 and 30 kPa and a pCO_2 _between 6.0 and 6.5 kPa. These sections were equilibrated for 30 minutes in oxygenated Krebs Henseleit Hepes (KHH) buffer containing (in mM): NaCl (118), KCl (4.8), NaHCO_3 _(27.2), MgCl_2 _(1.2), KH_2_PO_4 _(1.0), CaCl_2 _(1.20), glucose.H_2_O (10), HEPES (20) at a pH of 7.4 and a temperature of 37°C. The buffer was supplemented with 10% foetal calf serum (FCS: Harlanseralabs #S-0001A). Ischaemia was simulated by bubbling the media with 95% N_2 _and 5% CO_2 _in the absence of glucose (pH 6.6–6.9).

### Experimental protocols

#### Study 1: Effect of the intensity of ischaemic injury and the time of reoxygenation

After being equilibrated in oxygenated KHH buffer for 30 minutes, the atrial sections were randomly allocated to various protocols: 30, 90 and 180 minutes of ischaemia followed by 2, 8 or 24 hours of reoxygenation (n = 8/group). Some slices were not subjected to ischaemia and were aerobically incubated for 2, 8 or 24 hours to serve as aerobic controls. Creatine kinase (CK) release into the media was measured during the first 2 hours of reoxygenation and during the first 2 hours of aerobic incubation in the controls. The tissues were taken at the end of the protocols for the assessment of tissue viability, cell necrosis and apoptosis, and caspase activity (see below).

#### Study 2: The role of caspase activation in cell death

Free-hand sections of fresh atrial tissue were allowed to equilibrate in oxygenated KHH buffer for 30 minutes and then subjected to 90 minutes of simulated ischaemia followed by 120 minutes of reoxygenation (n = 4/group) in the absence (controls) and presence of 0.7, 7.0 and 70.0 nM concentrations of the specific caspase-3 inhibitor z.DEVD.FMK. The inhibitor was present in the media throughout the entire experimental protocol. The tissue was taken at the end of protocol and stored at -80°C until assessment of caspase-3 activity.

In an additional study, the atrial muscles (n = 6/group) were subjected to the following experimental protocols: (i) aerobic perfusion for 240 minutes; (ii) aerobic perfusion for 240 minutes with the caspase inhibitor z.DEVD.FMK (70 nM); (iii) 90 minutes simulated ischaemia followed by 120 minutes reoxygenation; and (iv) simulated ischaemia/reoxygenation with 70 nM z.DEVD.FMK (70 nM). The caspase inhibitor was incubated with the muscles for the entire experimental period. As before, the CK release was measured in the incubation media during the 120 minutes of reoxygenation or during the last 120 minutes of aerobic incubation in the controls and the tissue was taken at the end of protocols for the assessment of tissue viability and cell necrosis and apoptosis.

### Assessment of tissue injury and viability

CK release into the perfusate during the 2 hours of reoxygenation was measured as an index of tissue injury. The enzyme activity was measured by a linked-enzyme kinetic assay employing a commercial assay kit (DG147-K: Sigma Chemicals, Perth, Australia) and expressed as IU/g wet weight.

Tissue viability was assessed by the mitochondrial reduction of 3-[4,5 dimethylthiazol-2-y1]-2,5 diphenyltetrazolium bromide (MTT) to an insoluble purple formazan dye (M2128-Sigma Chemicals, Perth, Australia) at the end of the reoxygenation period, as previously described [26]. Finally, the absorbance of the blue formazan product was measured on a plate reader (Benchmark, Bio-Rad Laboratories, Hercules, CA, USA) at 550 nm and the results were expressed as mM of formazan/g wet weight.

### Assessment of apoptosis and necrosis

The muscles were incubated for 10 minutes on ice with 5 μM propidium iodide (PI) in 0.1 M tri-sodium citrate and 20 mM phosphate buffered saline (PBS) at pH7.4 to identify the necrotic nuclei. Sections were then fixed twice, first for 30 minutes then with 4% paraformaldehyde in 30% sucrose and 20 mM PBS overnight on ice and at pH7.4. Following this, serial sections of 10 μm were cut with a Bright cryomicrotome (model OTF) at -25°C in tissue embedding matrix (Tissue Tek^® ^OCT compound). Mirror sections were labelled at this stage with 20 μM PI to stain the total number of nuclei in each section. The cryopreserved tissue sections were washed with 20 mM PBS at pH 7.4 for 2 minutes, then permeabilised in 0.02 mg/ml proteinase-K for 10 min at 37Cs, and pre-sensitised for 1 minute in a microwave oven at 800 watts in 0.1% Triton X-100 and 0.1 M sodium citrate at pH 6.0. To assess apoptosis, the terminal deoxynucleotidyl transferase (TdT) was used to incorporate fluorescein (FITC) labelled dUTP oligonucleotides to DNA strand breaks at the 3'-OH termini in a template dependent manner (TUNEL technique) using a commercially available kit (Roche: 1684795, Basel, Switzerland). The FITC fluorescence emission (range 600–630 nm) was measured using argon-ion fluorescence excitation at 488 nm and detected by laser confocal epifluorescence microscopy with a ×10 oil immersion objective. The PI labelled nuclei was excited with helium-neon laser light at 543 nm and fluorescence was detected using an emission range of 680–730 nm in order to abolish fluorescence 'bleed-through' from FITC labelled nuclei. Analysis was done using NIH Image software (Scion Corp, Frederick, Maryland, USA) with the Cavalieri-3 macro (G. MacDonald, University of Washington). Fluorescent signals with areas greater than 16 μm^2 ^were counted to ensure that only cardiomyocyte nuclei are taken into account and to avoid the inclusion of artefact.

### Quantitation of caspase activity

The muscle sections stored at -80°C until analyses were thawed in 400 μl of cell lysis buffer (in mM: Hepes (100), 10% sucrose, 0.1% Chaps and DTT (10), in the presence of a cocktail of enzyme inhibitors (P2850-Sigma Chemicals, Perth, Australia) at a pH of 7.0 to release the intracellular contents. The sections were diced finely and then homogenised (Ultra-Turrax homogeniser: Janke and Kunkel GmbH, Staufen, Germany) at 13,000 rpm for 1 minute on ice. This was followed by centrifugation (PK121R-ALC International) at 14,000 rpm for 30 minutes. Subsequently, the protein concentration of the soluble supernatant (cell lysate) was measured using a detergent compatible Bio-Rad assay (23225-Pierce, Cheshire, UK). Aliquots of cell lysate were then tested for caspase activity by the addition of the caspase-specific substrate DEVD, that is conjugated to the chromophore (fluorescent reporter molecule) 7-amino-4-trifluoromethyl coumarin (AFC). The cleavage of the peptide DEVD from DEVD.AFC (final concentration 20 μM;Alexis Chemicals, San Diego, CA, USA) releases AFC that when excited by light at 400 nm emits fluorescence at 505 nm. The level of caspase activity in the cell lysate is detected by fluorescence signal obtained with a fluorometer (Fluostar P401, BMG software, Longmont, CO, USA). The amount of caspase-3 like activity was measured by using the effector caspase inhibitor z.DEVD.FMK at a final concentration of 10 μM in the well of the reader plate and by subtracting the fluorescence obtained in the presence of the inhibitor by the total fluorescence measured in the absence of the inhibitor. The inherent fluorescence of the aromatic residues in the protein homogenate was subtracted in the final calculations. The results were expressed as units of activity/g wet weight.

### Statistical analyses

Data were expressed as mean ± SEM. ANOVA was used for comparisons of means (Microsoft^® ^Excel analysis tool pak) with the application of a post-hoc Tukey's test. A p value of less than 0.05 was considered statistically significant.

## Authors' contributions

HAV participated in the design of the study, carried out all of the studies, collated and analyzed the data and drafted the manuscript. MG participated in the design of the study, analysis and presentation of the data and revisions of the manuscript. Both authors have read and approve the final manuscript.

## Figures and Tables

**Table 1:** CK leakage during the first 2 hours of reoxygenation of right atrial muscles (n = 8/group) subjected to various periods of simulated ischaemia followed by various periods of reoxygenation as compared with the enzyme leakage of muscles for the first 2 hours of aerobic incubation. *p < 0.05 vs the respective aerobic controls.

Reoxygenation group	Aerobic control	Simulated ischaemia (minutes)		
		30	90	180
2 hours	2.6 ± 0.2	3.5 ± 0.3*	5.0 ± 0.4*	6.4 ± 0.4*
8 hours	2.8 ± 0.3	4.0 ± 0.5*	5.0 ± 0.4*	6.6 ± 0.8*
24 hours	2.5 ± 0.4	3.7 ± 0.5*	4.6 ± 0.7*	6.1 ± 1.0*

**Table 2:** CK leakage during the first 2 hours of reoxygenation and MTT reduction at the end of the reoxygenation period of right atrial muscles (n = 6/group) after 90 minutes of simulated ischaemia/reoxygenation (SI/R) in the presence and absence of the caspase-3 inhibitor z.DEVD.FMK (70 nM).

Group	MTT	P value	CK	P value
Aerobic Control	290.3 ± 32.3		2.5 ± 0.2	
Aerobic Control plus C3i	298.0 ± 30.4	NS	2.7 ± 0.1	NS
SI/R	212.7 ± 23.1		5.4 ± 0.4	
SI/R plus C3i	220.3 ± 18.4	NS	5.7 ± 0.5	NS
